# Innovative digital technologies for quality assurance of diagnosis of human African trypanosomiasis

**DOI:** 10.1371/journal.pntd.0006664

**Published:** 2018-09-13

**Authors:** Epco Hasker, Jean Kwete, Raquel Inocencio da Luz, Alain Mpanya, Nicolas Bebronne, Jacquies Makabuza, Yves Claeys, Jérémie Ilunga, Veerle Lejon, Dieudonné Mumba Ngoyi, Philippe Büscher, Marleen Boelaert, Erick Mwamba Miaka

**Affiliations:** 1 Institute of Tropical Medicine, Department of Public Health, Antwerp, Belgium; 2 Programme National de Lutte Contre la Trypanosomiase Humaine Africaine, Kinshasa, the Democratic Republic of the Congo; 3 Institute of Tropical Medicine, Department of Biomedical Sciences, Antwerp, Belgium; 4 Institut de Recherche pour le Développement, University of Montpellier, Montpellier, France; 5 Institut National de Recherche Biomédicale, Department of Parasitology, Kinshasa, the Democratic Republic of the Congo; Institut de recherche pour le developpement, FRANCE

## Policy platform

Early diagnosis and treatment is a key and often life-saving strategy in many disease control programs, including those for human African trypanosomiasis (HAT). Good quality diagnosis is hence as important as good quality medicine, though quality assurance (QA) systems in the diagnostics field tend to be less well developed [1]. In recent years, the reported number of HAT cases has drastically decreased, and the World Health Organisation aims to eliminate HAT due to *Trypanosoma brucei gambiense* as a public health problem by 2020. In this context, there is a need to strictly maintain the quality of diagnostic procedures, both for screening and for confirmation of HAT diagnosis [2]. We discuss the specific challenges of QA systems for HAT diagnosis, compare them with QA systems for diagnostics in tuberculosis (TB) control, and highlight possible solutions making use of new technologies.

A minimal QA program comprises of quality control (QC), external quality assessment (EQA), standard operating procedures (SOP), and competency assessment (CA) of coworkers. To achieve certification by the International Organization for Standardization (ISO), the ISO 15189:2012 regulation specifies requirements for quality and competence in medical laboratories [3]. QC is an internal system focusing on intrinsic performance of tests used, quality of reagents and materials used, and inclusion of positive and negative controls. CA probes the ability of laboratory staff to properly execute their tasks and can be evaluated through internal quality assurance (IQA) systems, such as reprocessing a number of samples and comparing results [4]. The central concept of EQA is to monitor the level of performance of clinical laboratories by comparing them to a national or supranational standard. The EQA systems in use in disease control programs are often—but not always—based on the dispatching of a "proficiency panel," i.e., a set of well-characterized samples with a known result prepared by a reference laboratory and sent for reanalysis and rereading to the clinical laboratories. One possible downside of EQA based on testing of proficiency panels is that it monitors the optimal performance of a laboratory but not necessarily its routine performance level [5].

TB control programs relying on sputum smear microscopy for diagnosis have for many years understood the need for permanent QA systems. Both IQA and EQA systems are in use. The recommended system of EQA for Ziehl–Neelsen staining relies on a re-examination of slides from each laboratory involved in the program [6]. All slides have to be stored, and periodically, a laboratory supervisor will take a random sample of slides and dispatch it to the reference lab for rechecking. The results of the expert reader are then compared to those of the clinical lab. A major asset of this EQA system is that it monitors routine rather than optimum performance. A Lot Quality Assurance Sampling procedure (LQAS) is used to ensure that, with minimum effort, all laboratories in the network are monitored with comparable sensitivity and specificity [7]. The actual number of slides to be sampled depends on the workload of the peripheral laboratory and the proportion of positive slides reported [8]. Finding false positive slides or more than the allowed number of false negatives will trigger further investigation. Such systems have proven their value and ensure the reliability of TB microscopy results in many countries [9].

**Fig 1:**
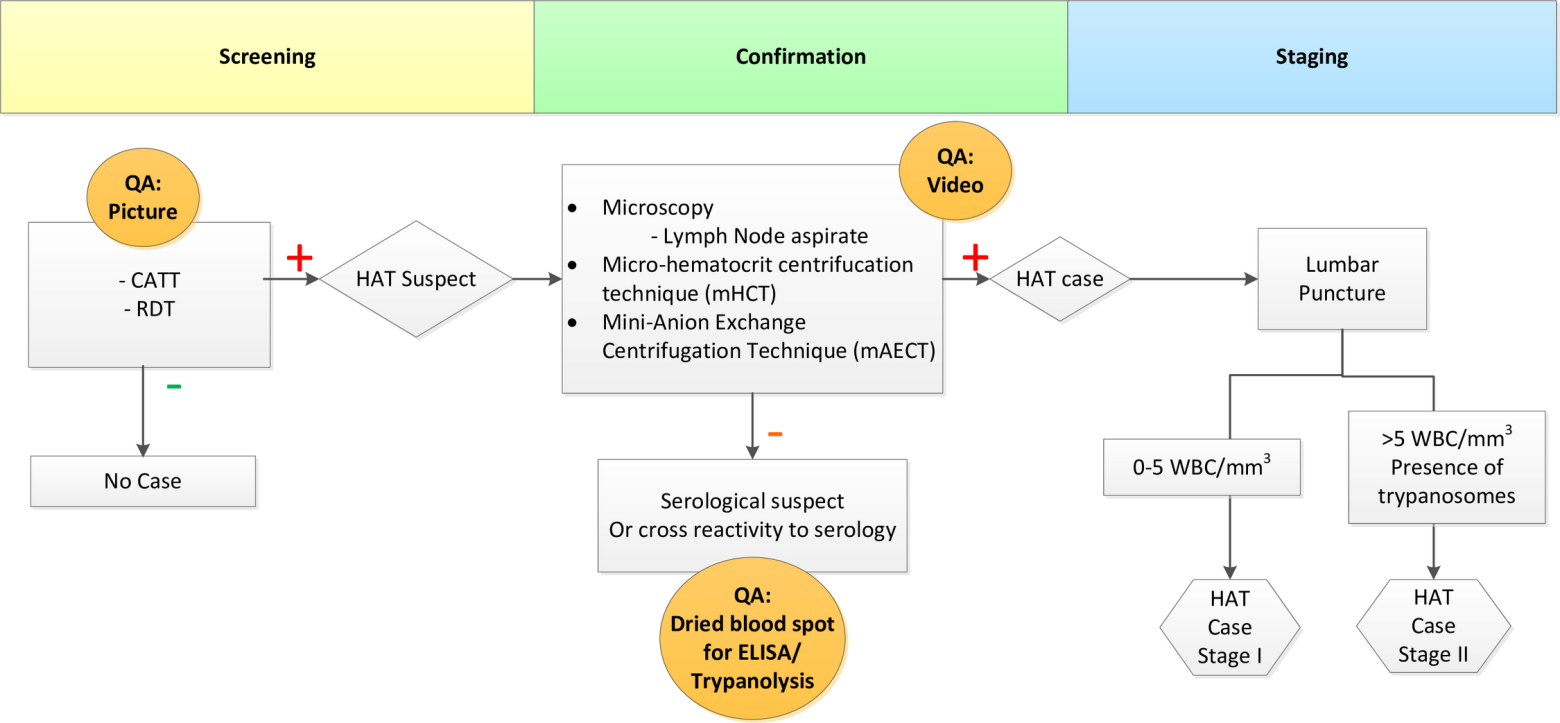
The diagnostic algorithm for HAT in use in DRC and potential implementation of digital quality control systems [18]. DRC, Democratic Republic of the Congo; HAT, human African trypanosomiasis.

The development of a QA system for gambiense–HAT diagnosis has always posed major challenges. The diagnostic algorithm for HAT involves two steps: first, a screening by an antibody detection test such as the card agglutination test for trypanosomiasis (CATT)/*T*.*b*. *gambiense* or a Rapid Diagnostic Test (RDT) to identify suspects, followed by a set of confirmation tests, usually the direct microscopic examination of lymph node puncture aspirate and/or Giemsa-stained thick blood film, followed by a concentration method on blood such as the microhematocrit centrifugation technique (mHCT) or the mini-Anion Exchange Centrifugation Technique (mAECT) (Fig 1). In these concentration methods, living parasites can be seen at relatively low magnifications. The live parasites make characteristic movements, and for many years, the diagnostic procedure has been considered to be virtually 100% specific if performed well. Unfortunately for EQA considerations, preparations of live parasites (such as mAECT) cannot be stored. This severely limits the options for EQA. Microscopy slides of thin or thick films stained with Giemsa can be stored, but these are not the preferred ones for routine diagnosis of HAT because of their poor sensitivity. The same limitations that apply to storing slides would apply to proficiency panels being sent out by a reference lab. Because of these obvious constraints, HAT control programs have so far only focused on IQA.

HAT control programs conduct case detection in two different contexts: 1. active screening sessions conducted at the community level by mobile units and 2. passive detection in fixed health centers. In active screening campaigns, IQA is assured by assigning two readers to each sample and having a third senior reader solve the discrepancies. In passive screening, usually no IQA is implemented so far.

In the context of HAT elimination initiative, even stricter QA rules apply while increasing its complexity. The sensitivity of parasitological confirmation tests in HAT has always been an issue. Even optimal combinations of all available tests do not reach more than 95% sensitivity in most evaluations [10–12]. This inherent deficit in sensitivity, in a context of declining HAT incidence, easily leads to eroding laboratory skills. Between October 2013 and April 2014, an ad hoc EQA assessment with a focus on malaria diagnosis was conducted in the Democratic Republic of the Congo (DRC), targeting fixed laboratories in the public and private sector. Based on a standard set of five stained blood slides (thick films and thin films) dispatched to 445 laboratory technicians, it was observed that, overall, less than half of the participants (45.4%) could correctly report the presence of *Trypanosoma*. This number went further down to 16.5% when the slide manifested a mixed *Plasmodium falciparum*/*Trypanosoma* spp. infection. Recognition of *Trypanosoma* was significantly better in participants who declared to routinely perform the diagnosis of HAT compared to those who did not perform the diagnosis of HAT routinely (22% versus 8%), but even in the former category, performance was still poor. Also, an assessment of routinely processed thick blood films in those laboratories showed that only 13.6% were properly prepared and stained [13].

**Fig 2:**
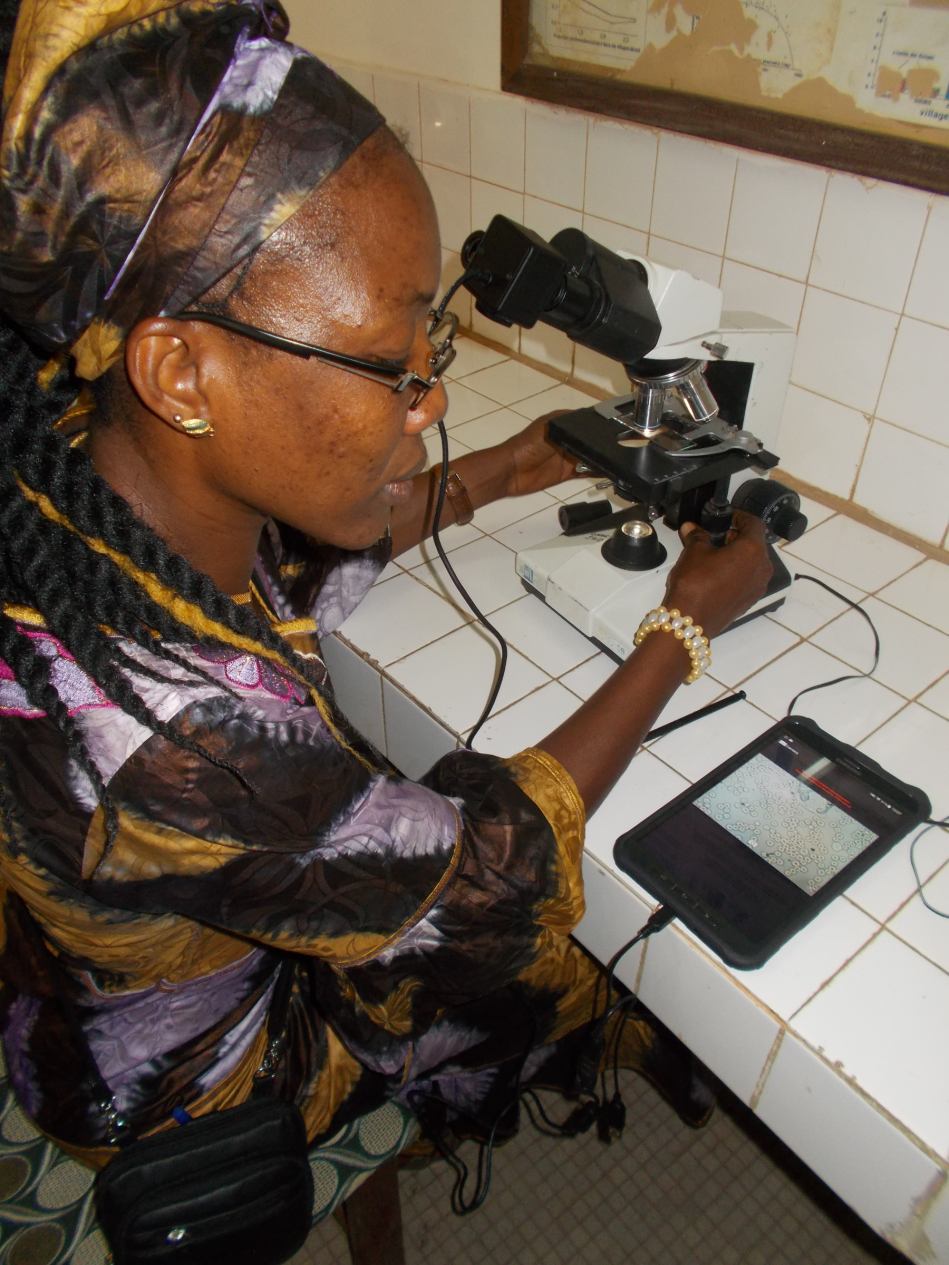
Digital imaging system for recording videos of microscopic images using an android tablet and external camera mounted onto the ocular tube of a microscope.

In recent years, as HAT is increasingly becoming a rare disease, there may also be a problem of false positive HAT diagnoses, as observed in the northeast of DRC [14]. This is where digital innovation may step in and become a game changer. A recent pilot study conducted by the national HAT control program of the DRC (PNLTHA) and the Institute of Tropical Medicine (ITM) showed that it was technically possible to record a short video of the moving trypanosomes in the mAECT collector tube. Using a smartphone application developed by our team, the microscopy technician was able to record an image of any positive confirmation test. We fit the smartphone on the trinocular microscope with a custom-designed adapter. This allows recording a video through the lens of the microscope, making use of the camera of the smartphone. In a preliminary evaluation of 114 CATT and/or RDT positive HAT suspects in Bandundu province examined in their villages by a lab technician, two were confirmed as HAT cases. For both patients, good quality videos of the parasite were recorded, which could be rechecked at a distance by a senior reader, leaving no doubt about the diagnosis. Blood samples on filter paper were obtained for all 114 HAT suspects and tested in the laboratory with immune trypanolysis, a highly sensitive and specific test for HAT antibodies [15]. Two samples out of 114 tested positive, which corresponded to the samples of the two HAT patients identified by the lab technician in the village. Though a sample size of two confirmed cases is insufficient to draw far-reaching conclusions on the performance of the system, the potential offered by digital tools is obvious. This potential could be exploited in a well-designed EQA system, adapted to the specific context. In the meantime, we have further developed the video tool by developing an application that can be used on any Android 5 smartphone or tablet and that uses an external camera fitted in the tube of the ocular, making the methodology more robust and obliterating the need for a trinocular microscope (Fig 2). The app ensures that the recorded videos are automatically saved with the registration number of the patient and with a timestamp for easy and foolproof linkage to the corresponding patient record. This innovative digital imaging system allowed us to overcome the issue of not being able to store positive *T*.*b*. *gambiense* slides. The same app also addresses EQA of the screening tests (CATT or RDT), as a picture of the screening test can be taken and stored for later rechecking by the supervisor. Synchronization of the data in the tablet or smartphone with a central database on a network associated server (NAS) occurs whenever a WiFi connection is made.

At least equally important in EQA is a system to ensure that samples read as negative are truly negative. Here, TB control programs recognize that errors may occur because a technician will never be able to examine each and every field in a slide. He will examine 100–200 fields, which are not necessarily the same fields as those examined by the expert in the reference lab. Thus, it may happen that false negative slides do occur, and depending on the threshold chosen in the LQAS approach, a certain number of false negatives may be accepted [6]. In HAT, recording videos of negative diagnostic confirmation tests would be meaningless. What can be done, however, is to collect blood samples on filter paper of all subjects on whom diagnostic confirmation tests are done, as was done in the pilot study mentioned earlier. Such filter papers can be stored at ambient temperature in zipper bags containing silicagel to be sent to a reference lab on a regular basis. At the reference lab, these samples can be tested with enzyme-linked immunosorbent assay (ELISA), immune trypanolysis, or a sequence of both tests. As in the TB EQA system, finding more than the expected number of positives among supposedly negative samples does not necessarily equate poor performance but should trigger further investigation, e.g., a visit to the lab concerned by a laboratory supervisor.

Whereas in TB control, microscopy is usually performed in fixed health facilities that are part of the general healthcare system, as explained above; microscopy for HAT is also performed by specialized mobile teams that are part of a vertical program. In the DRC, there are currently 35 such teams. The EQA system described above can easily be applied to such mobile teams as well. It will enable the HAT control program management to focus its limited supervisory and training capacity on those teams that need it most. As HAT is increasingly becoming a rare disease, it is also important to bear in mind the lessons learned in the early days of TB control programs. When Styblo tried to fully integrate TB control into primary healthcare, he found out that microscopy was not always performed in a reliable manner (personal communication, Jaap Veen). Some health workers just did not have enough exposure to acquire the skills required. Styblo then advised to differentiate the tasks, meaning some health centers would refer suspect cases and others would provide the full diagnostic workup [16, 17]. A similar approach will be required for HAT as well because one cannot expect a health worker seeing no more than one HAT case per year to maintain adequate skills in HAT microscopy. To put in place a well-organized EQA system in the facilities that currently conduct microscopy does not have to cost much more than what is already spent. The Android tablet and external camera used in our projects together cost approximately US$600, which is not much compared to the cost of a good microscope. On the other hand, access to mobile phones and internet network is currently still challenging; until network coverage is complete across the country, creative solutions, such as offline data collection and uploading at a later stage, are feasible.

In conclusion, QA in HAT diagnostics is needed and is technically feasible. New digital technologies offer possibilities that were, until recently, not there to set up a comprehensive permanent QA system. The next step will be to test the operational feasibility and effectiveness of these tools at a larger scale and in routine working conditions.
